# Kaempferol Protects Against Amyloid β Overproduction and the Rise of Phospho-Tau 217 and Phospho-Tau 181 in the Rat *Cerebellum* Induced by Acute 3-Nitropropionic Acid Administration

**DOI:** 10.3390/ijms27062880

**Published:** 2026-03-22

**Authors:** Virginio García-López, Carmen López-Sánchez, Joana Poejo, Ricardo Lagoa, Dorinda Marques-da-Silva, Virginio García-Martínez, Carlos Gutierrez-Merino

**Affiliations:** 1 Department of Medical and Surgical Therapeutics, Division of Clinical Pharmacology, School of Medicine, University of Extremadura, 06006 Badajoz, Spain; garcialopez@unex.es; 2 Department of Human Anatomy and Embryology, Faculty of Medicine and Health Sciences, Institute of Molecular Pathology Biomarkers, University of Extremadura, 06006 Badajoz, Spain; virginio@unex.es; 3 Institute of Molecular Pathology Biomarkers, University of Extremadura, 06006 Badajoz, Spain; joanapoejo86@gmail.com; 4 School of Technology and Management, Polytechnic Institute of Leiria, Morro do Lena, Alto do Vieiro, 2411-901 Leiria, Portugal; ricardo.lagoa@ipleiria.pt (R.L.); dorinda.silva@ipleiria.pt (D.M.-d.-S.); 5 LSRE-LCM and ALiCE–Associate Laboratory in Chemical Engineering, Faculty of Engineering, University of Porto, Rua Dr. Roberto Frias, 4200-465 Porto, Portugal

**Keywords:** 3-nitropropionic acid, kaempferol, *cerebellum*, A1-type astrocytes, amyloid β, phosphorylated tau, antihemorrhagic action

## Abstract

The 3-nitropropionic acid (NPA) promotes neurological alterations in the *striatum*, *hippocampus* and vicinal motor and pre-motor cortical areas, and in the *cerebellum*. The neurological alterations induced by systemic NPA administration resemble those found in Huntington’s disease. In previous works, we have shown that intraperitoneal (i.p.) administration of kaempferol can efficiently protect against *striatum* degeneration and against motor neurological dysfunctions induced by NPA. In this work, we show that i.p. administration of kaempferol also protects against the increase in pro-inflammatory cytokines that potentiate the activation of complement C3 protein (a biomarker of A1-type reactive astrocytes generation) and overproduction of neurotoxic amyloid β (Aβ) peptides in the *cerebellum* of rats treated with acute i.p. administration of NPA. In NPA-treated rats, large multipolar neurons of cerebellar *nuclei* and Purkinje neurons of the cerebellar *cortex* are the cells that are most intensely stained by anti-C3 and by anti-Aβ antibodies. In addition, we found that kaempferol also protects against the NPA-induced increase in phospho-tau 217 and phospho-tau 181 in the *cerebellum*, and our results pointed out that the NPA-induced phospho-tau 217 colocalizes with Aβ(1-42) more closely than phospho-tau 181, both in dentate *nucleus* and cerebellar *cortex*. Also, our results unveil another novel brain-protective action of i.p. kaempferol co-administration: namely, its ability to prevent microhemorrhages induced in the cerebellar *nuclei* area by acute NPA administration. In conclusion, the results of this work show a potent protection of kaempferol against the NPA-induced increase in degeneration biomarkers in the *cerebellum*.

## 1. Introduction

The chemical 3-nitropropionic acid (NPA), produced by some fungi that infest plants [1,2,3], is an irreversible inhibitor of mitochondrial succinate dehydrogenase [4]. In rodents and non-human primates, the strong *striatum* degeneration and neurological alterations elicited by systemic NPA administration resemble those found in Huntington’s disease (HD) [4,5]. The fact that systemic NPA administration also produced memory impairment in rodents [6] suggests that this neurotoxin affects other brain regions. Also, cognitive dysfunction, visuospatial deficits, memory loss, and difficulty in learning new skills have been reported in the pre-motor stages of HD [7,8]. Indeed, NPA administration has been shown to promote neurological alterations in the *hippocampus* and vicinal motor and pre-motor cortical areas, and in the *cerebellum* [9,10,11,12]. It is worth noting that *striatum* and motor and premotor brain cortex establish functional connections with cerebellar *nuclei*, in particular with the dentate *nucleus* [13,14,15]. Although the *striatum* is the brain structure that is more extensively damaged at shorter times after NPA administration [4,5,10], NPA-induced activation of neuroinflammatory microglia [16,17,18] and nuclear factor kappa B (NF-κB) activation [12,16,17,18] contribute to spreading neurodegeneration to other brain areas. This leads to enhanced secretion of pro-inflammatory cytokines [16,17,18] and the subsequent rise in reactive oxygen species and nitric oxide production in the brain [19], which has also been shown to mediate NPA-induced brain neurodegeneration [4,10,20,21].

Kaempferol, a flavonol present in vegetables and fruits used in human nutrition, is a natural antioxidant which also inhibits the production of proinflammatory cytokines [22,23]. In previous works, we have shown that intraperitoneal (i.p.) administration of kaempferol can efficiently protect against *striatum* degeneration and against motor neurological dysfunctions induced by the acute i.p. administration of NPA [10,12,24]. Also, intravenous administration of kaempferol affords an efficient protection against damage of the *striatum*, elicited by transient focal cerebral ischemia, another brain insult associated with early mitochondrial dysfunction [25]. Because of kaempferol’s low toxicity to humans, this compound seems to be a good candidate for its therapeutic use in the treatment of NPA intoxications. Remarkably, therapeutic applications of kaempferol with particular emphasis on its anti-inflammatory effects have already been suggested [23,26,27].

Interleukin-1α (IL-1α), tumor necrosis factor α (TNFα), and complement component 1q (C1q), cytokines which are secreted by activated microglia, acting together are necessary and sufficient to induce the generation of the highly neurotoxic reactive A1-type astrocytes [28]. Complement C3 protein gene expression is highly upregulated in reactive A1 astrocytes, and is a specific biomarker of reactive A1 astrocytes generation [28]. We have shown that chronic systemic administration of NPA induces the generation of reactive A1-type astrocytes in the *striatum*, *hippocampus*, and *cerebellum* of rat brains [11]. Of note, in post-mortem samples of HD, abundant reactive A1 astrocytes have been identified [28], as well as cerebellar *cortex* damage with extensive Purkinje cells loss [29]. More recently, we showed that kaempferol i.p. administration prevented the proteolytic activation of complement C3 protein induced by acute systemic administration of NPA in the *striatum* and *hippocampus* [12].

Several pro-inflammatory cytokines upregulate the expression of amyloid β precursor protein (APP) in human neuroblastoma cells and non-neuronal cells like human astrocytes in culture, and also in the mouse brain [30]. Indeed, it is now recognized that reactive astrocytes can produce neurotoxic amyloid β (Aβ) peptides [30,31]. Furthermore, A1-like astrocytes, which are derived from U251 human astroglioma cells by treatment with a mixture of TNFα, IL-1α and C1q, show increased production of APP and Aβ peptides [32]. In a previous study [12], we found that i.p. acute NPA administration potentiates the production of neurotoxic Aβ peptides in the more severely affected rat brain structures, namely, in the *striatum* and in the *hippocampus*, and that kaempferol coadministration fully prevented this effect of NPA. These results are relevant when taking into account that NPA induces tau pathology in tangle-mouse model and in wild-type [33], and that the neurotoxic Aβ(1-42) peptide has been reported to form complexes with tau, promoting self-aggregation and potentiating tau phosphorylation [34]. Therefore, the possibility that kaempferol coadministration may prevent the overproduction of neurotoxic Aβ peptides and of phosphorylated tau by NPA deserves to be studied. To this end, the rat *cerebellum* constitutes a better choice than the *striatum* and *hippocampus*, because of the high tissue damage induced by acute NPA administration in the latter brain structures.

In this work, we have experimentally assessed the effect of acute i.p. NPA administration and kaempferol co-administration in adult Wistar rats’ *cerebellum*, following the criteria and protocols described in our previous publications. Also, we have identified the cerebellar structures that display the highest increase in pro-inflammatory cytokines, neurotoxic Aβ(1-42) and selected phospho-Tau species.

## 2. Results

### 2.1. Kaempferol i.p. Co-Administration Affords an Efficient Protection Against NPA-Induced Damage in the Cerebellum

We have shown previously that the i.p. co-administration of kaempferol+NPA (KNPA) affords an efficient protection against the damage induced by acute i.p. administration of NPA in the *striatum*, *hippocampus* and vicinal cortical motor areas of the rat brain [10,12].

**Figure 1:**
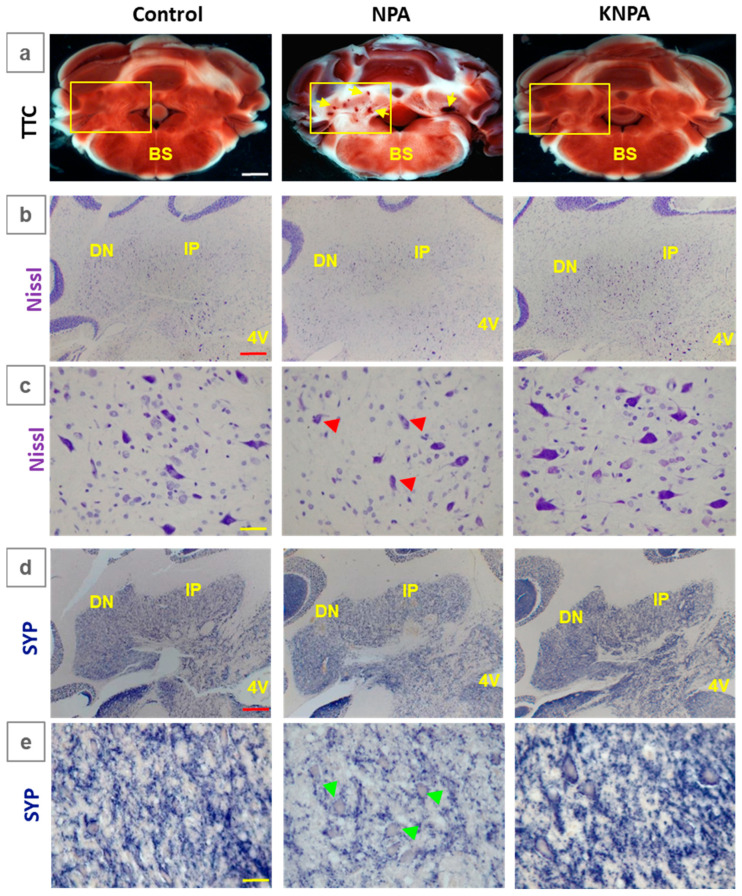
Kaempferol protects against NPA-induced damage to the *cerebellum*. Representative coronal sections of the *cerebellum* of the control, NPA and KNPA groups after staining with 2,3,5-triphenyltetrazolium chloride (TTC), Nissl staining and immunohistochemistry with anti-synaptophysin (SYP). Panel (**a**) illustrates the white areas and microhemorrhages (yellow arrows) induced by NPA treatment detected in the cerebellar *nuclei* (NPA group) with TTC staining. The damaged tissue is undetectable in rats treated with kaempferol (KNPA group), showing a TTC staining intensity that was similar to the control group. The yellow square mark indicates the area of the *cerebellum* shown in panels (**b**–**e**). Note the decreased Nissl staining in the soma of cerebellar *nuclei* neurons in the NPA group compared with the control and KNPA groups in panel (**b**). Higher magnification images of Nissl staining (**c**) show the decrease in large multipolar neurons (red arrows) in the dentate *nucleus* (DN), which is completely prevented by concomitant treatment with kaempferol. Comparative immunostaining with anti-SYP (**d**,**e**) shows synaptic destruction in the cerebellar *nuclei* after treatment with NPA and their preservation with kaempferol co-administration. Note the decrease in synaptic vesicles in the dentate *nucleus* neurons (green arrows) of the NPA group in the high magnification images (**e**). BS: brain stem; IP: interposed *nucleus*; and 4V: fourth ventricle. White scale bar: 2 mm. Red scale bar: 200 μm. Yellow scale bar: 25 μm.

Our results reveal that co-administration of KNPA afforded a nearly complete protection against NPA-induced damage in the *cerebellum*, as assessed using TTC, Nissl, H&E, and synaptophysin immunostaining (Figure 1 and Figure S1). As shown in Figure 1a, TTC staining reveals that NPA induced significant damage of cerebellar *nuclei*, including fastigial, mainly in the interposed and dentate *nucleus*. Significantly, TTC and H&E staining showed an efficient protection of kaempferol against NPA-induced damage in these cerebellar areas. Indeed, the images of NPA-treated rats pointed out the occurrence of microhemorrhages in this region of the *cerebellum* and that these were not seen in the images of KNPA rats (Figure 1a and Figure S1). On the other hand, Nissl staining, which labels the endoplasmic reticulum, which is mainly associated with somas in neurons [35], decreased by less than 20% of the intensity in the NPA-treated group (Figure 1b). By using higher magnification (Figure 1c), Nissl staining of the dentate *nucleus* revealed around a 50% decrease in the neuronal somas of large multipolar neurons, which is completely prevented by kaempferol co-treatment. Moreover, the staining with anti-synaptophysin antibody, which is specifically associated with synapses linked to vesicle secretion [36], showed a more significant intensity decrease in NPA-treated rats with respect to the control rats, particularly in the dentate *nucleus*, and that kaempferol completely protected against the decrease in the NPA-induced intensity of anti-synaptophysin immunostaining (Figure 1d). Additionally, the quantitative analysis of the higher magnification images of the dentate *nucleus* (Figure 1e) yielded a decrease of around 35% in staining intensity with respect to the control rats. These results pointed out that the NPA treatment produced a significant loss of neuronal connections in this neuronal structure of the *cerebellum*. Also, the results indicate that the co-treatment with kaempferol affords protection against NPA-induced neuronal damage in these areas.

### 2.2. Kaempferol i.p. Co-Administration Prevents the Increase in Proinflammatory Cytokine Levels and the Generation of Reactive A1 Astrocytes Induced by NPA in the Cerebellum

**Figure 2:**
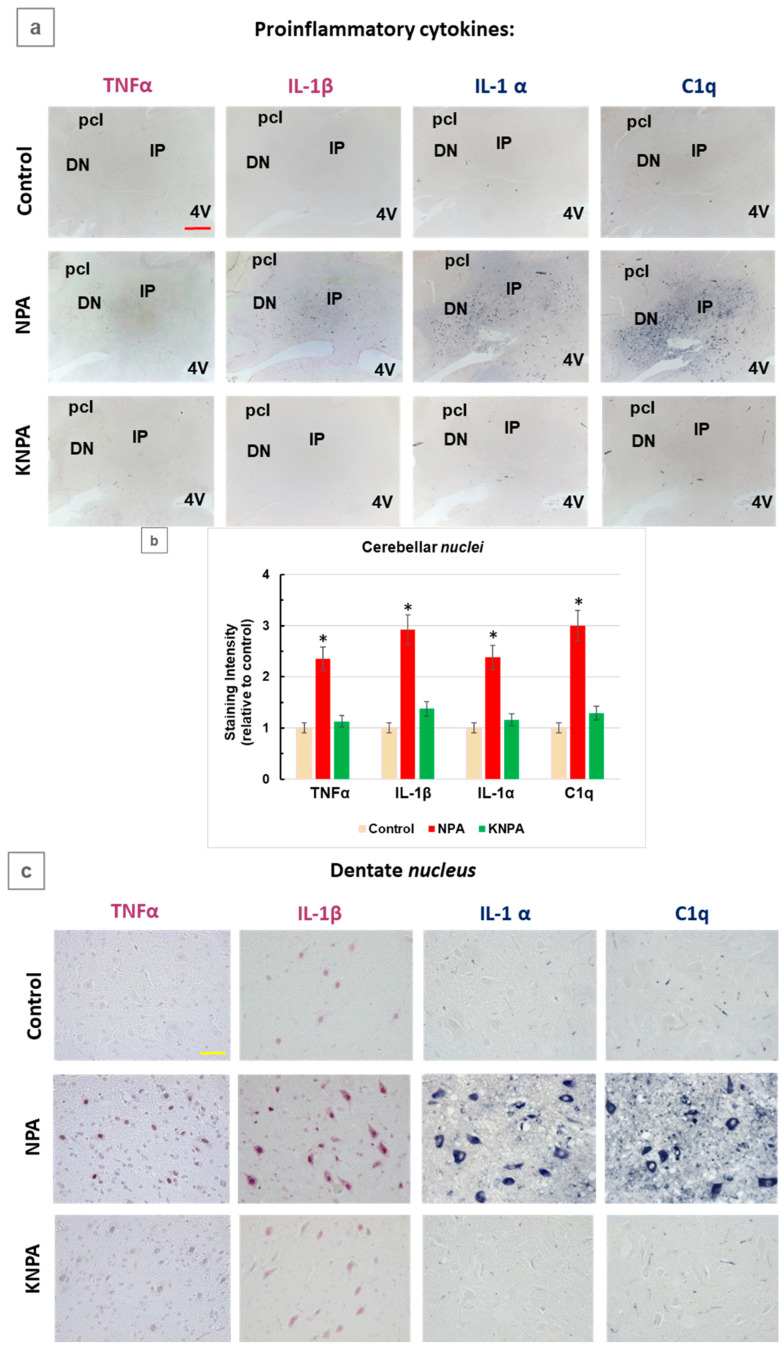

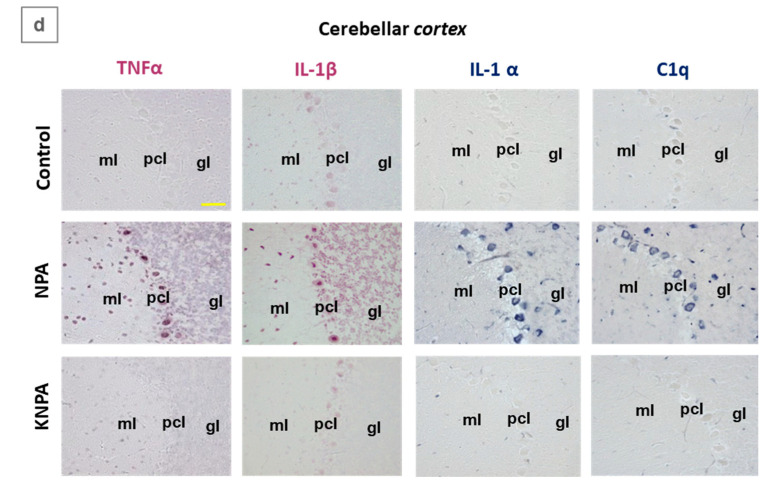
Kaempferol prevents an increase in proinflammatory cytokines TNFα, IL-1β, IL-1α and complement C1q in the *cerebellum* of NPA-treated rats. (**a**) Representative coronal sections of the cerebellar *nuclei* after immunohistochemistry, with anti-TNFα, anti-IL-1β, anti-IL-1α and anti-C1q corresponding to the control, NPA and KNPA groups. The NPA group shows the intense immunoreactivity of these cytokines, both in the interposed (IP) and dentate *nuclei* (DN). (**b**) The mean values of the intensity of positive pixels in TNFα, IL-1β, IL-1α, and C1q staining images, from *n* = 3 different rats in each group, obtained with ImageJ^®^ software. The asterisks (*) indicate that the intensity of positive pixels is significantly higher in the NPA group, with respect to the control group: *p* = 0.0009 and CI = 1.9–2.7 for TNFα, *p* = 0.0004 and CI = 2.4–3.4 for IL-1β, *p* = 0.0007 and CI = 2.0–2.8 for IL-1α, and *p* = 0.0004 and CI = 2.5–3.5 for C1q. Cerebellar nuclei sections of rats treated with kaempferol (KNPA group) show a low immunoreactivity, similar to that of the control group. Higher magnification images of the dentate *nucleus* (**c**) and cerebellar cortex (**d**) show somas of large multipolar neurons of the dentate *nucleus* and Purkinje cells stained with all four cytokines in the NPA group. No staining in the cellular soma is observed in the control and KNPA groups. Note that the images of the dentate *nucleus* stained with anti-IL-1α and anti-C1q antibodies (**c**), which also stain small blood vessels, reveals NPA-induced thickening of microvasculature in this region of the *cerebellum*. Abbreviations: 4V: fourth ventricle; pcl: Purkinje cell layer; ml: molecular layer; and gl: granular layer. Red scale bar: 200 μm. Yellow scale bar: 25 μm.

It is well-known that the brain inflammation caused by proinflammatory cytokines mediates the neurodegenerative process induced by NPA. In this work, we have focused on TNFα, IL-1α and C1q, which are the cytokines that elicit the generation of A1-type neurotoxic reactive astrocytes [11,28], as well as interleukin-1β (IL-1β), which has been shown to foster neurotoxic Aβ peptides production [37,38]. Immunohistochemistry with specific antibodies of cerebellar slices revealed significant changes in the expression levels of the selected proinflammatory cytokines in cerebellar slices of control rats, NPA and KNPA (Figure 2). The direct visual inspection of the results showed the overall increase in the level of these cytokines induced by NPA in the cerebellar *nuclei*, and that this increase is largely attenuated by the i.p. co-treatment with kaempferol (Figure 2a). As indicated in detail in Section 4, using ImageJ^®^ software, we evaluated the staining intensity after threshold correction by the subtraction of the background intensity (Figure 2b). These results pointed out that NPA induced a two- to three-fold increase in TNFα, IL-1β, IL-1α and C1q, and, also, that the co-treatment with kaempferol prevented the increase in the expression level of these cytokines. The images shown in Figure 2c also revealed that the somas of large multipolar neurons in the dentate *nucleus* account for most of the NPA-induced increase in staining intensity with antibodies against these specific cytokines. In addition, as anti-IL-1α and anti-C1q antibodies also stain small blood vessels [39,40], the images of the NPA group of Figure 2c also show that NPA increases the level of proinflammatory cytokines surrounding small blood vessels.

Furthermore, analyzing the cerebellar *cortex* (Figure 2d), we observed that the Purkinje cell layer is the most heavily stained with antibodies that are specific for the selected cytokines in rats of the NPA group. Additionally, we observed significant differences in the pattern of the rise of these cytokines in the cerebellar cortex induced by NPA, i.e., the NPA-induced increase in TNFα and IL-1β in the granular cell layer is much higher than the NPA-induced increase in the other two cytokines: namely, IL-1α and C1q. Overall, the results of the KNPA group revealed the strong protection afforded by kaempferol co-treatment against the increase in the expression level of these cytokines in the cerebellar *cortex* as well.

**Figure 3:**
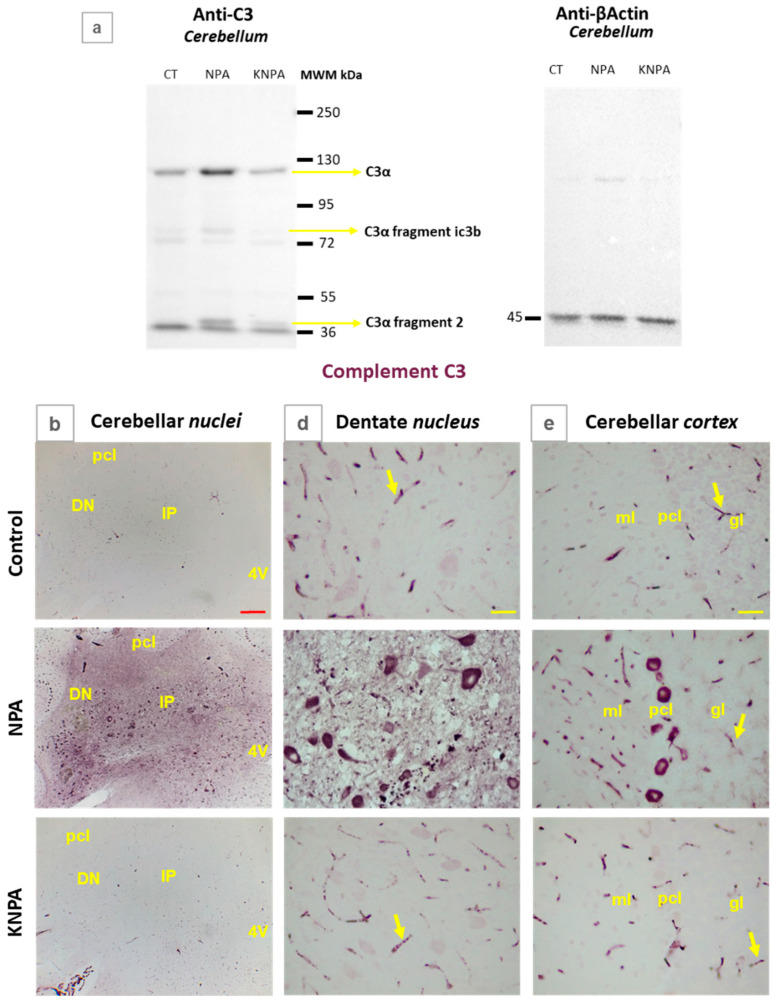

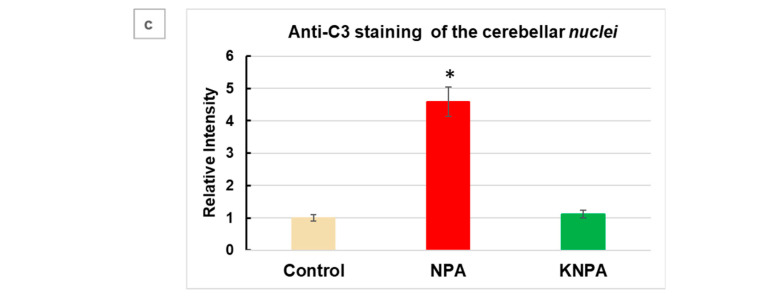
Kaempferol protects against NPA-induced complement C3 activation in the *cerebellum.* (**a**) Representative Western blots of C3 protein and β-actin of *cerebellum* homogenates of rats in the control group (CT), NPA group (NPA) and KNPA group (KNPA). After acquisition of images of the Western blot with anti-C3, the PVDF membrane was stripped and processed for the Western blot of anti-β-actin. Kaempferol protects against the increase in C3α and C3α proteolytic fragments induced by the administration of NPA in the *cerebellum*, with respect to rats of the CT. (**b**) Representative coronal sections after immunohistochemistry with anti-C3, corresponding to the cerebellar *nuclei* of the control, NPA and KNPA groups. Rats treated with NPA (NPA group) show intense C3 immunoreactivity in both the interposed (IP) and dentate *nuclei* (DN). (**c**) Mean values of the intensity of positive pixels of the images of C3 staining, from *n* =3 different rats of each group obtained with ImageJ^®^ software. The intensity of C3 immunostaining is significantly higher in the NPA group compared to the control group (*), *p* = 0.0002 and CI = 3.85–5.35. Rat *cerebellum* sections treated with kaempferol (KNPA group) show a low immunoreactivity, which is not significantly different to the control group. Higher magnification images of the dentate *nucleus* (**d**) and cerebellar *cortex* (**e**) show that in the NPA group, the somas of large multipolar neurons of the dentate *nucleus* and Purkinje cell layer are the most intensely stained with anti-C3 antibody. A significant characteristic of anti-C3 antibody is that it also stains small blood vessels in the cerebellar slices, which are marked with yellow arrows in the three groups. Abbreviations: 4V: fourth ventricle; pcl: Purkinje cell layer; ml: molecular layer; gl: granular layer. Red scale bar: 200 μm. Yellow scale bar: 25 μm.

Next, we experimentally evaluated the level of activated complement C3 protein in the *cerebellum* of the three rat groups, a specific marker used in neurodegenerative disorders [11,41,42]. The results are shown in Figure 3. As can be observed in Figure 3a, Western blots demonstrate that the level of active C3 complement protein fragment C3α (the strong protein band), C3α fragment iC3b and C3α fragment 2 significantly increase in NPA-treated rats, relative to the control rats. Also, these results show that co-treatment with kaempferol almost completely prevents the NPA-induced increase in these active C3 complement protein fragments. The quantification of the C3α protein band/β-actin ratio yielded a 2.1-fold increase with respect to the control rats, which was found to be significant (*p* = 0.0012). Thus, these results point out the activation of the complement C3 protein in NPA-treated rats. In addition, the quantification of the Western blot lane of co-treatment with kaempferol and NPA allows us to conclude that kaempferol afforded more than 95% protection against the NPA-induced activation of the complement C3 protein.

We also experimentally assessed the expression level of active C3 complement protein fragments in the *nuclei* of the *cerebellum* and cerebellar *cortex* by using immunohistochemistry (Figure 3b–e). We observed the highest staining intensity in the cerebellar *nuclei* region in cerebellar slices of the NPA group (Figure 3b), as quantified by the intensity analysis of this region (Figure 3c). These results demonstrate that NPA induces an increase in active C3 complement protein in the cerebellar *nuclei* region and that this increase is more than 90% attenuated by kaempferol co-treatment, in good agreement with the results obtained by Western blotting. The NPA-induced increase in active C3 complement protein levels determined by immunohistochemistry images is higher than in Western blotting, 4.6-fold versus 2.1-fold, which is consistent with a stronger activation of the C3 complement protein in cerebellar *nuclei* than in other areas of the *cerebellum*. Of note, the highest levels of anti-C3 staining are displayed in large multipolar neurons of the dentate nucleus (Figure 3d) and Purkinje cell layer of the cerebellar *cortex* (Figure 3e).

Besides the activation of C3 protein, which is a reactive A1 astrocytes marker [11,28], gliosis appears during NPA-induced brain neurodegeneration [10]. Therefore, we also experimentally assessed that the NPA treatment induces gliosis in the cerebellar regions studied herein, using immunohistochemistry with anti-GFAP (Supplementary Figure S2). The results showed a nearly two-fold increase in the anti-GFAP staining intensity in NPA-treated rats compared with control rats. It should be noted as well that co-treatment with kaempferol affords more than 90% attenuation of the increase in staining with anti-GFAP induced by NPA.

**Figure 4:**
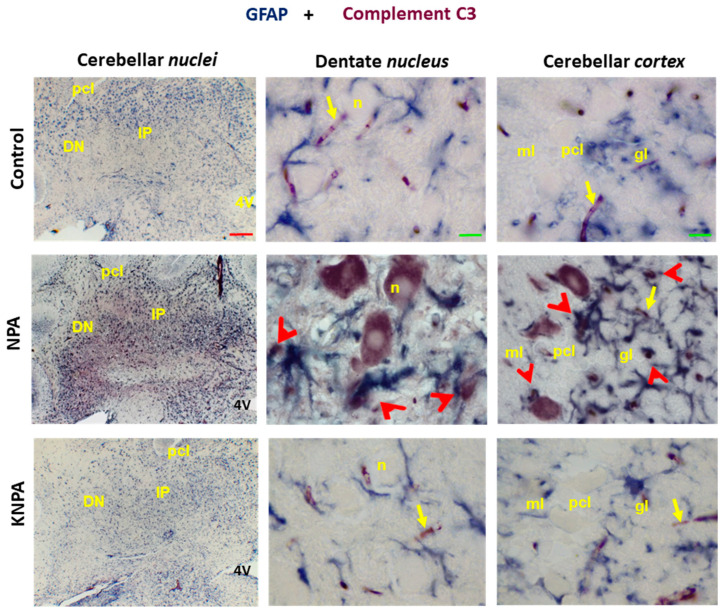
Kaempferol prevents the generation of reactive A1 astrocytes induced by NPA in the *cerebellum.* Double immunohistochemistry with anti-GFAP (in blue) and anti-C3 (in red) in coronal sections of the cerebellar *nuclei* prepared from the control, NPA and KNPA groups. In the NPA group, higher magnification images of the dentate *nucleus* (DN) and cerebellar *cortex* show large somas of multipolar neurons (n) of the dentate *nucleus* and Purkinje cells, stained with C3, and co-location of GFAP and complement C3 protein in ameboid-shaped reactive A1 astrocyte (red arrowheads). C3 staining or co-location of GFAP and complement C3 protein is not observed in the control and KNPA groups. Of note, small blood vessels intensely stained with the anti-C3 antibody are marked in the three groups with yellow arrows. Abbreviations: 4V: fourth ventricle; pcl: Purkinje cell layer; ml: molecular layer; gl: granular layer. Red scale bar: 200 μm. Green scale bar: 10 μm.

Taking this into account, we performed double immunochemistry experiments with anti-C3 and anti-GFAP (Figure 4). Immunostaining with anti-C3α and anti-GFAP is revealed by red and blue, respectively. Our results show, again, the C3 complement protein location in large neurons of the cerebellar *nuclei* and Purkinje cells of the cerebellar *cortex*, while double labeling shows overlap in the staining with both antibodies in vicinal large-sized astrocytes surrounding the somas of large multipolar neurons of cerebellar *nuclei* and glial cells located around the Purkinje cells’ soma. This is further highlighted by the analysis of selected microscopy images of large cells present in the dentate *nucleus* and cerebellar *cortex* with the split channel tool of ImageJ^®^ (Supplementary Figure S3), obtained as indicated in Section 4.5.3. Brighter pixels correspond to those showing a higher intensity of staining with anti-GFAP (blue channel) and with anti-C3 (red channel). Note that colocalization of C3 complement protein and GFAP cannot be observed in the microscopy images of the KNPA group, nor in those of the control rats, due to the very weak staining of these samples with these antibodies.

### 2.3. Kaempferol i.p. Co-Administration Prevents the Increase in the Aβ(1-42) Levels Induced by NPA in the Cerebellum

**Figure 5:**
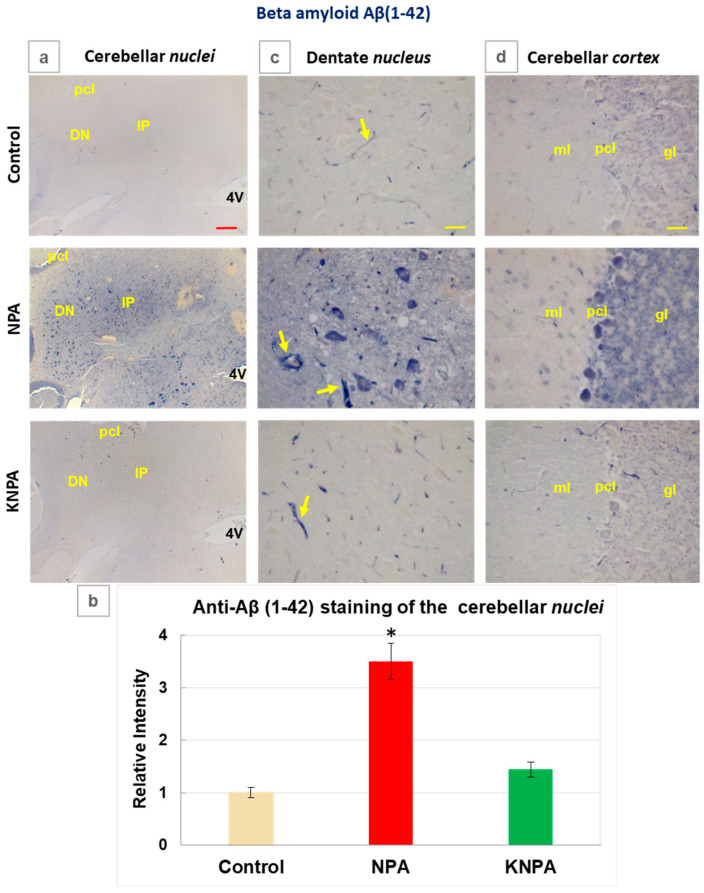
Kaempferol prevents the increase in the Aβ(1-42) levels induced by NPA in the *cerebellum.* (**a**) Representative coronal sections after immunohistochemistry with anti-beta amyloid Aβ(1-42), corresponding to the cerebellar *nuclei* of the control, NPA and KNPA groups. Rats treated with NPA (NPA group) show intense Aβ(1-42) immunoreactivity in both the interposed (IP) and dentate *nuclei* (DN). (**b**) The mean values of the intensity of positive pixels of the images of Aβ(1-42) staining, from *n* = 3 different rats of each group obtained with ImageJ^®^ software. The intensity of Aβ(1-42) immunostaining is significantly higher in the NPA group compared to the control group (*), *p* = 0.0003 and CI = 2.4–4.1. Rat *cerebellum* sections treated with kaempferol (KNPA group) show a low immunoreactivity, which is not significantly different to that found for the control group. Higher magnification images of the dentate *nucleus* (**c**) and cerebellar *cortex* (**d**) show large somas of multipolar neurons of the dentate *nucleus*, as well as the Purkinje and granular layers that were strongly stained with Aβ(1-42) in the NPA group. A significant characteristic of anti-Aβ(1-42) antibody staining of cerebellar slices is that it intensely stains small blood vessels (marked in the three groups with yellow arrows). Abbreviations: 4V: fourth ventricle; pcl: Purkinje cell layer; ml: molecular layer; and gl: granular layer. Red scale bar: 200 μm. Yellow scale bar: 25 μm.

Reactive A1 astrocytes can produce Aβ(1-42) [32,38] and, also, it has been shown that IL-1β potentiates the production of neurotoxic Aβ peptides through the amyloidogenic pathway [37,38]. Since our results revealed the increase in both the reactive A1 astrocytes and the proinflammatory cytokine IL-1β in NPA rats, we have experimentally evaluated the levels of Aβ(1-42) using immunohistochemistry (Figure 5). Staining of *cerebellum* slices revealed that Aβ(1-42) levels increased around 3.5-fold in the cerebellar *nuclei* region of NPA-treated rats, with respect to the control rats (Figure 5a,b). Interestingly, the co-administration of kaempferol reduced the NPA-induced increase in Aβ(1-42) levels by more than 80% in the cerebellar *nuclei*. The anti-Aβ(1-42) immunohistochemistry images obtained with a higher resolution (Figure 5c,d) show that large multipolar neurons of the dentate *nucleus* and Purkinje cells layer of the cerebellar *cortex* display the highest level of anti-Aβ(1-42) staining. Also, the images of the cerebellar *cortex* point out an approximately two-fold rise in the intensity of anti-Aβ(1-42) staining in the granular cell layer in NPA-treated rats relative to the control rats, which is fully prevented by co-administration of kaempferol (Figure 5d). Congo Red staining of cerebellar slices (Supplementary Figure S4), a qualitative histological method used for identifying amyloid plaques and tangles [43], gives additional experimental support to the anti-Aβ(1-42) immunohistochemistry results.

**Figure 6:**
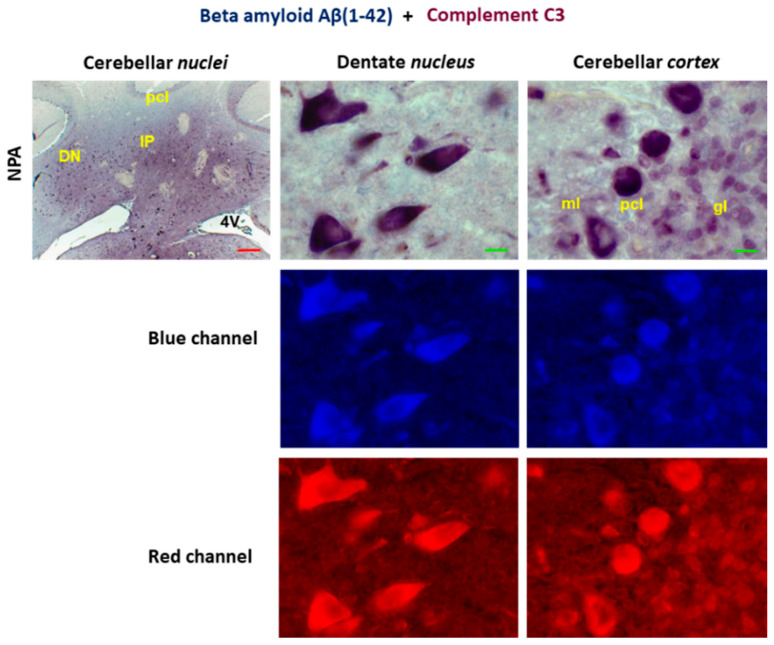
Co-localization of Aβ(1-42) and complement C3 in the *cerebellum* of the NPA group. Double immunohistochemistry with anti-Aβ(1-42) (in blue) and anti-C3 (in red) in coronal sections of cerebellar *nuclei* prepared from the NPA group. Higher magnification images of the dentate *nucleus* and cerebellar *cortex* show co-localization of Aβ(1-42) and complement C3 protein in large somas of multipolar neurons of the dentate *nucleus* and in the Purkinje cells layer (pcl). As shown by the analysis of the selected microscopy images with the split channel tool of ImageJ^®^ software, there is an extensive co-localization of anti-Aβ(1-42) and anti-C3 staining. Abbreviations: 4V: fourth ventricle; ml: molecular layer; and gl: granular layer. Red scale bar: 200 μm. Green scale bar: 10 μm.

Since our results revealed that complement C3 increases in NPA rats both in neuronal somas and reactive A1 astrocytes, by means of double immunohistochemistry, we analyzed the staining pattern with anti- Aβ(1-42) and anti-C3 in *cerebellum* slices. We observed an overlap in the staining with both antibodies in cerebellar *nuclei* and Purkinje cell layer of the cerebellar *cortex* (Figure 6). This is further highlighted by the analysis of selected microscopy images using the split-channel tool of the ImageJ^®^ software, performed as indicated in the Materials and Methods (Section 4.5.3). Brighter pixels correspond to those showing a higher intensity of staining with anti-Aβ(1-42) (blue channel) and with anti-C3 (red channel). The visual inspection of the blue and red images highlights a close spatial relationship between the activation of complement C3 protein and the enhanced production of Aβ(1-42) in the *cerebellum* of NPA-treated rats. The densitometric histograms of the blue and red channel images revealed that the intensity of the background signal (darker pixels) is less than 10% of that of the brighter pixels, pointing out a minor contribution of unspecific labeling to the intensity of the latter pixels.

### 2.4. Kaempferol i.p. Co-Administration Prevents the Increase in Phospho-Tau 217 and Phospho-Tau 181 Levels Induced by NPA in the Cerebellum

**Figure 7:**
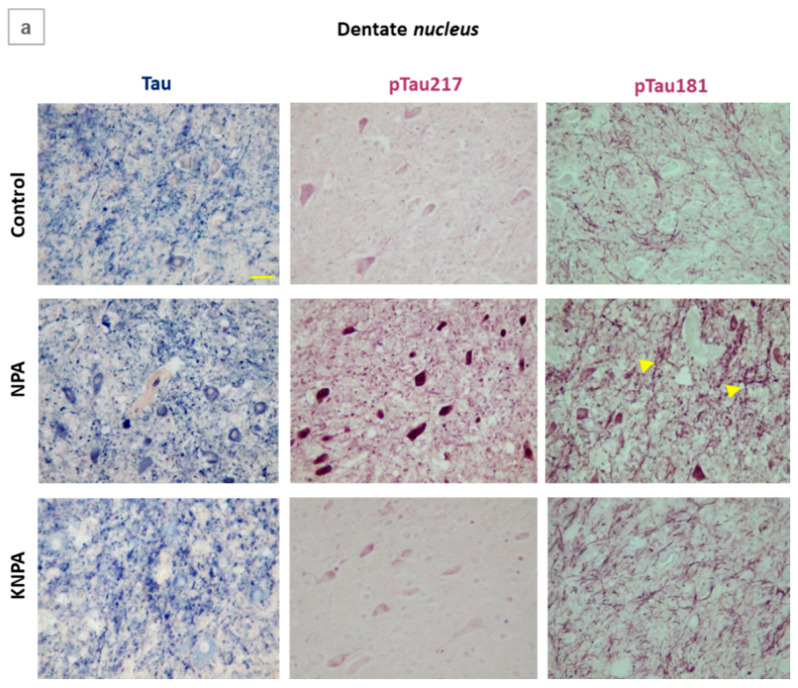

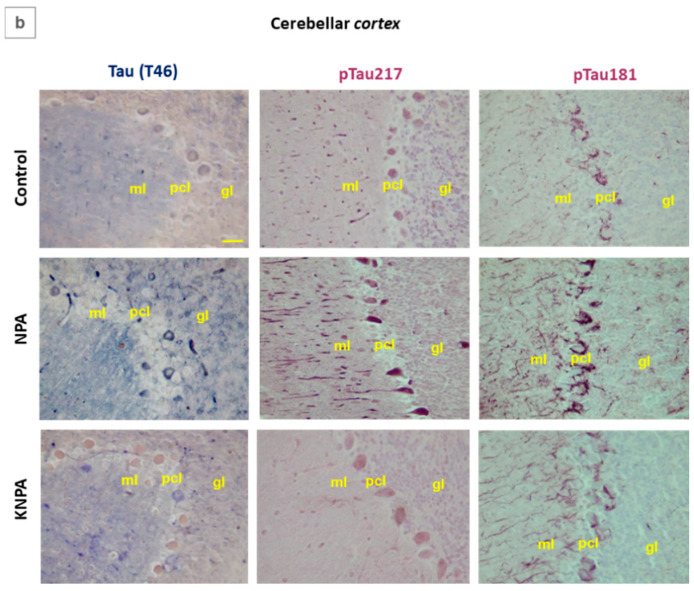
Kaempferol prevents the increases in tau phosphorylation in the *cerebellum* of NPA-treated rats. Representative coronal sections of dentate *nucleus* (**a**) and cerebellar *cortex* (**b**) after immunohistochemistry with anti-Tau, anti-phospho-Tau Thr217 (pTau217) and anti-phospho-Tau Thr181 (pTau181), corresponding to the control, NPA and KNPA groups. The NPA group shows increased levels of these markers. In panels (**a**,**b**), coadministration of kaempferol shows that it almost completely prevents both structural alterations in cell morphology and the increased staining of Tau, pTau217 and pTau181 antibodies, displaying an intensity of staining that is not significantly different to that of the control group. Note the marked differences in the staining pattern observed with pTau217 and pTau181 antibodies in the cerebellar areas of NPA treated rats: pTau217 intensely stains the large neuronal cell bodies of the dentate *nucleus* (**a**) and the cell bodies of Purkinje neurons (**b**), whereas pTau181 stains both the large somas of multipolar neurons of the dentate *nucleus* and the processes (yellow arrows) extending from the reactive A1 astrocyte (**a**) more intensely, as well as in the processes of Bergmann glial cells surrounding the soma of Purkinje cells in the cerebellar *cortex* (**b**). pcl: Purkinje cell layer; ml: molecular layer; and gl: granular layer. Yellow scale bar: 25 μm.

It is known that neurotoxic Aβ(1-42) peptide forms complexes with tau, enhancing tau phosphorylation [34]. Furthermore, phospho-tau 217 and phospho-tau 181 have been proposed as biomarkers of tau hyperphosphorylation, detected at the early stages of brain degeneration in Alzheimer’s disease, a well-established tauopathy [44]. It has also been reported that NPA induces tau pathology in both the tangle-mouse model and wild-type mice [33]. On these grounds, by using immunohistochemistry with specific antibodies, we experimentally evaluated tau, phospho-tau 217 and phospho-tau 181 levels in *cerebellum* slices. Our results revealed increased levels of these markers in rats in the NPA group (Figure 7). There are large differences in the staining pattern observed with phospho-tau 217 and phospho-tau 181 antibodies in the areas of the *cerebellum* of NPA-treated rats. In the case of dentate *nucleus* (Figure 7a), anti-phospho-tau 217 stains the soma of large multipolar neurons more intensely, while anti-phospho-tau 181 stains the thick neuronal extensions more intensely, likely monitoring NPA-induced axonal degeneration. In the cerebellar *cortex* (Figure 7b), anti-phospho-tau 217 stains the soma of Purkinje cells and the thick dendritic extensions likely protruding from these cells more intensely, while anti-phospho-tau 181 stains the Bergmann glial cells that colocalize around the Purkinje cells soma more intensely. Also, the staining pattern with the anti-tau antibody points out that the NPA treatment induces significant structural alterations in the cellular morphology of the dentate *nucleus* and the *cortex* of the *cerebellum*, which is in good agreement with the results shown above for anti-synaptophysin staining. Notably, co-administration of kaempferol (rats of the KNPA group, Figure 7) efficiently protects against the NPA-induced rise in phospho-tau 217 and phospho-tau 181.

**Figure 8:**
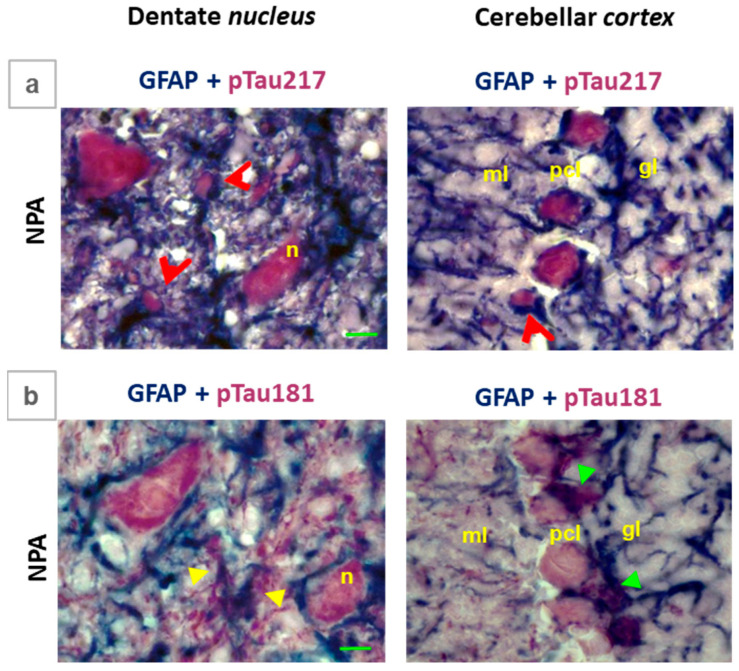
Comparative colocalization of GFAP with pTau217 and GFAP with pTau181 in the *cerebellum* of the NPA group. Selected microscopy images corresponding to the NPA group of large cells present in the dentate *nucleus* and cerebellar *cortex* after double labeling immunohistochemistry using anti-GFAP with anti-pTau217 (**a**), as well as anti-GFAP with anti-pTau181 (**b**). Panel (**a**) shows an intense labeling with anti-pTau217 (red color) in the neuronal soma of large multipolar neurons (n) of the dentate *nucleus* and of Purkinje neurons of the cerebellar *cortex*, which is clearly segregated from the labeling with anti-GFAP. Note the colocalization of GFAP and pTau217 in ameboid-shaped reactive A1 astrocytes (red arrowheads). Panel (**b**) illustrates the overlap between pTau181 (red color) and GFAP (blue color) staining in processes extending from reactive A1 astrocytes (yellow arrows) and in the processes of Bergmann glial cells surrounding the soma of Purkinje cells in the cerebellar *cortex* (green arrows). pcl: Purkinje cell layer; ml: molecular layer; and gl: granular layer. Green scale bar: 10 μm.

In order to further assess the contribution of glial cell staining, we performed double labeling immunohistochemistry in the *cerebellum* slices by using anti-GFAP with anti-phospho-tau 217, as well as anti-GFAP with anti-phospho-tau-181 (Figure 8). Our results show an intense labeling with anti-phospho-tau 217 (red color) in the neuronal soma of large multipolar neurons in the dentate *nucleus* and of Purkinje neurons in the cerebellar *cortex* (Figure 8a), which is clearly segregated from the labeling with anti-GFAP (blue color). In contrast (Figure 8b), the labeling with anti-phospho-tau 181 shows a more diffuse pattern between extensions of large multipolar neurons (red color) and glial cells labeled with anti-GFAP (blue color). Of note, the overlap between anti-phospho-tau 181 and anti-GFAP staining is particularly strong in the intensely stained Bergmann glial cells that colocalize around the Purkinje cells soma (Figure 8b).

**Figure 9:**
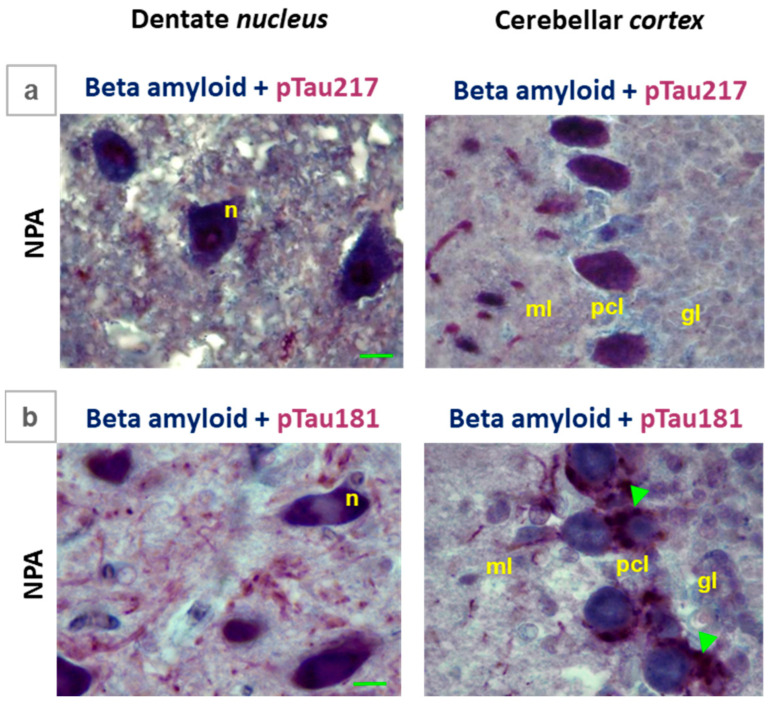
Comparative colocalization of Aβ(1-42) with pTau217 and Aβ(1-42) with pTau181 in the *cerebellum* of the NPA group. Selected microscopy images corresponding to the NPA group of large cells present in the dentate *nucleus* and cerebellar *cortex* after double labeling immunohistochemistry using anti-Aβ(1-42) with anti-pTau217 (**a**), as well as anti-Aβ(1-42) with anti-pTau181 (**b**). Note that pTau217 colocalizes with Aβ(1-42), both in dentate *nucleus* and cerebellar *cortex*, more closely than pTau181. Panel (**a**) shows a clear overlap of anti-Aβ (blue) and anti-pTau217 (red) staining in the neuronal soma of large multipolar neurons (n) in the dentate *nucleus* and the soma of Purkinje cells in the cerebellar *cortex*. Panel (**b**) shows a more segregated staining pattern between anti-pTau181 (red) and anti-Aβ (blue). Note a clear segregation between anti-Aβ, which stains the Purkinje cell soma, and anti-pTau181, which exclusively stains the processes of Bergmann glial cells (green arrows) surrounding the Purkinje cell soma, in the cerebellar *cortex*. In the dentate *nucleus*, the overlap between the staining with both antibodies is only evident in the soma of large multipolar neurons. pcl: Purkinje cell layer; ml: molecular layer; and gl: granular layer. Green scale bar: 10 μm.

Due to the relevance of the interactions between Aβ(1-42) and hyperphosphorylated tau for the seeding and potentiation of the formation of neurofibrillary tangles [34,45], we performed double labeling immunohistochemistry of the *cerebellum* slices with anti-Aβ/anti-phospho-tau 217 and anti-Aβ/anti-phospho-tau 181 (Figure 9). Our observations revealed a clear overlap of the labeling with anti-Aβ (blue color) and anti-phospho-tau 217 (red color) in the intensely stained neuronal soma of large multipolar neurons of the dentate *nucleus* and of the Purkinje cells soma of the cerebellar *cortex* (Figure 9a). In contrast (Figure 9b), the labeling with anti-phospho-tau 181 (red color) shows a more clearly distinct pattern than that of anti-Aβ (blue color); albeit, overlap between the labeling with both antibodies is clear in the soma of large multipolar neurons of the dentate *nucleus*. In the cerebellar *cortex*, an extensive overlap between anti-Aβ and anti-phospho-tau 181 labeling can be observed in the Bergmann glial cells, which lie close to the Purkinje cells soma.

## 3. Discussion

The results of this work demonstrate that i.p. co-administration of kaempferol efficiently prevents the rise in proinflammatory cytokines TNFα, IL-1α, C1q and IL-1β induced by acute i.p. administration of NPA in the rat *cerebellum*. It should be recalled that these cytokines have been shown to foster A1-type reactive astrocytes generation [28] and neurotoxic Aβ peptides overproduction [32,37,38]. Moreover, it has been shown that A1-type astrocytes can also contribute to the production of Aβ(1-42) [32,38].

The almost complete blockade of the NPA-induced rise in active C3 complement protein (a biomarker of A1-type reactive astrocytes [28]) by kaempferol bears a special relevance, because C3 complement protein activation is required for neurodegeneration in mouse models of amyloidosis and tauopathy [46]. Of note, this action of kaempferol is consistent with the reported inhibition of the complement system activation by kaempferol-rich flavonoid extracts from medicinal plants [47]. Also, our results support that the protection of kaempferol against overproduction of neurotoxic Aβ peptides is likely the cumulative effect of the attenuation and eventual blockade of NPA-induced increase in proinflammatory cytokines, and of A1-type reactive astrocyte generation in the *cerebellum*.

Our results point out that the protective effects of kaempferol are particularly strong in the large neuronal somas of the cerebellar *nuclei* and in the Purkinje cells layer of the cerebellar *cortex*. Interestingly, an isocoumarin analog (kaempferol isomer), which improves neuronal outcomes through the modulation of tropomyosin kinase B receptor signaling, has been proposed to have therapeutic potential for the treatment of various disorders of the central nervous system [48]. This bears a special relevance, due to the major role of large multipolar neurons of the cerebellar *nuclei* in the control and coordination of motor movements, which are impaired in NPA-treated rats [10,11,12]. In this regard, it is worth recalling that the dentate *nucleus* has important neuronal functional connections with the motor and premotor brain neocortex, cortical areas that undergo more damage in the NPA-induced brain degeneration [10,11]. Of note, while acute i.p. administration of NPA induces a large damage in the rat brain areas of the *striatum* and *hippocampus* (with noticeable tissue disaggregation and extensive cell loss [10,12]), the tissue damage in the *cerebellum* is less severe, i.e., small microhemorrhages and thickening of microvascular vessels can be seen in the cerebellar *nuclei* area of NPA-treated rats. Since an excess of inflammatory mediators production may cause irreversible vascular damage leading to insufficient tissue perfusion, organ dysfunction and death [49], this vascular damage could, at least in part, be mediated by NPA-induced overproduction of proinflammatory cytokines. Indeed, in NPA-treated rats, blood vessels of the cerebellar *nuclei* are heavily stained by the anti-C3 antibody and, also, by the anti-Aβ(1-42) antibody (Supplementary Figure S5). It should be recalled that cerebral amyloid angiopathy is characterized by the deposition of Aβ in blood vessels [50]. In contrast, no indications of microhemorrhages or of capillaries thickening are seen either in the control or in (kaempferol+NPA)-treated rats, a finding that is consistent with kaempferol’s reported ability to inhibit vascular endothelial inflammation and to exert other protective effects on the vascular *endothelium* [26]. Therefore, these results unveil another novel brain-protective action of i.p. kaempferol co-administration: namely, its beneficial brain antihemorrhagic action. This beneficial effect of kaempferol appears to be promising and warrants further development in a separate study focused on NPA-induced brain vascular pathology.

Besides the pathophysiological relevance of the functional impairment of the cerebellar *nuclei,* mentioned above in the neurological dysfunctions induced by acute NPA intoxication, it should be remarked that the extent of tissue damage in this brain area is clearly lower than that produced in the *striatum* and *hippocampus* by the same treatment with NPA, analyzed in detail in our previous works [10,12]. Nevertheless, it must be noted that Nissl staining and the immunohistochemistry with anti-synaptophysin and with tau reveal significant neuronal and structural alterations, as well as the loss of synaptic connections in the cerebellar nuclei, particularly in the dentate *nucleus*. Therefore, the results shown in this work point out that *cerebellum* degeneration induced by acute i.p. administration of NPA is somewhat delayed with respect to that of the *striatum*, *hippocampus* and vicinal motor and premotor areas of the brain *cortex*. In spite of the fact that the degeneration of the *cerebellum* induced by acute i.p. administration of NPA is at an initial stage relative to that of the *striatum* and *hippocampus*, NPA induced the rise in proinflammatory cytokines, gliosis and activation of C3 complement protein in the *cerebellum*. This gives further experimental support to our previous conclusion that the generation of A1-type reactive astrocytes is an early event in the neurotoxicity induced by the chronic administration of NPA [11]. Recently, it has been shown that the astrocyte leaflets enwrap many neuron synapses in clusters, integrating synaptic activity at a circuit functional level [51]. Since the disruption of astrocyte leaflets foster neurological degeneration, the progressive degeneration and loss of functional axonal connections between the cerebellar *nuclei* and the above-mentioned brain areas could play a significant role to potentiate the onset of NPA-induced degeneration in the cerebellar *nuclei*. Also, it has been noted that pro-inflammatory mediators secreted by reactive astrocytes can induce disruption of tight junctions, finally leading to blood–brain barrier integrity breakdown and brain edema [52,53]. Interestingly, several works have shown beneficial actions of kaempferol to preserve the blood–brain barrier’s integrity [54,55]. Furthermore, in a cerebral ischemia–reperfusion rat middle cerebral artery occlusion model, kaempferol has been shown not only to preserve the blood–brain barrier’s integrity, but also to inhibit neutrophils activation, aggregation and infiltration into the brain [56]. Let us recall that disruption of the blood–brain barrier potentiates the infiltration of neutrophils from the blood circulation into the brain, worsening the brain injury outcome because this increases morbidity and mortality in cerebral ischemia–reperfusion [57]. Infiltrating neutrophils produce pro-inflammatory cytokines, matrix metalloproteinases, nitric oxide, reactive oxygen species and other cytotoxic molecules that accelerate brain damage in the ischemic brain [58].

Our microscopy images of NPA-induced increases in the different cytokines studied highlighted that the most intensely stained cells are the large neuronal somas of the cerebellar *nuclei* and the Purkinje cells layer of the cerebellar *cortex*. Interestingly, the same conclusions are reached from the double immunohistochemistry microscopy images stained with the anti-C3 and anti-Aβ(1-42) antibodies. It should be recalled that the gene encoding the amyloid precursor protein displays a widespread transcription in rat brain neurons, with high levels in several brain areas: in particular, in Purkinje cells and cerebellar granule cells [59]. Later, it was remarked that intracellular Aβ(1-42) is significantly higher in Purkinje neurons of rats’ *cerebellum* than in granule neurons of the cerebellar *cortex* [60]. Our microscopy images stained with anti-GFAP and anti-C3 pointed out an extensive overlap between the staining afforded with both antibodies, suggesting that A1-type astrocytes are likely the main responsible for the increase in C3 levels. Since the double immunohistochemistry microscopy images stained with anti-Aβ(1-42) and anti-C3 also showed an extensive overlap between the staining afforded with both antibodies, our results show that activation of the C3 complement protein correlates with the increase in Aβ(1-42) levels induced by NPA in the *cerebellum*. Although further experimental studies are needed to demonstrate a causal relationship between A1-type astrocytes generation and Aβ(1-42) overproduction in NPA-induced brain degeneration, this hypothesis is supported by the fact that i.p. coadministration of kaempferol elicits a nearly complete blockade of NPA-induced activation of C3 complement protein in the *cerebellum*, and more than 80% attenuation of the increase in neurotoxic Aβ peptides. It is worthy to mention herein that herbal extracts of *Persicaria lapathifolia* containing kaempferol glycosides inhibit the classical pathway of C3 complement protein activation [61]. Nevertheless, it should be noted that the microscopy images of the cerebellar *cortex* stained with anti-Aβ(1-42) showed that NPA also induced a significant widespread increase in the Aβ(1-42) level in the granular cell layer, an increase that is not observed in the microscopy images of the cerebellar *cortex* stained with anti-C3. In contrast, the spatial pattern in the NPA-induced increase in the staining intensity of the granular cell layer with TNFα and IL-1β looks similar to the spatial pattern of the increase observed with anti-Aβ(1-42). As these cytokines have been shown to foster the amyloidogenic processing of the amyloid β precursor protein [37,38], it seems plausible that this can account, at least in part, for the observed NPA-induced increase in neurotoxic Aβ peptides in the granular cell layer of the *cerebellum*.

The results of this work highlight that kaempferol i.p. administration has a strong protective effect against the NPA-induced overproduction of neurotoxic Aβ peptides in the rat *cerebellum*. It is worth noting here that the neurotoxic Aβ(1-42) peptide has been reported to form complexes with tau, promoting self-aggregation and potentiating tau phosphorylation [34,45] and that NPA induces a tau pathology in the tangle-mouse model and in wild-type mice [33]. Our results show that kaempferol co-administration elicits almost complete attenuation of the NPA-induced increase in phospho-tau 217 and phospho-tau 181, which are phospho-tau species reported to be detected at the early stages of brain degeneration in Alzheimer’s disease, a well-established tauopathy [44,62,63]. Therefore, our results show that kaempferol i.p. administration can also be useful to protect against NPA-induced tauopathy. In this regard, it should be noted that the results of this work pointed out that the NPA-induced phospho-tau 217 colocalizes with Aβ(1-42), both in the dentate *nucleus* and cerebellar *cortex*, more closely than phospho-tau 181. Note that the double immunohistochemistry microscopy images show a clear segregation between the labeling with anti-phospho-tau 217 and with anti-GFAP, while the labeling with anti-phospho-tau 181 overlap with glial cells stained with anti-GFAP. Indeed, anti-phospho-tau 217 heavily stains the large neuronal somas of the dentate *nucleus* and the somas of Purkinje neurons, which is in excellent agreement with the results obtained with anti-Aβ(1-42) antibody staining. In contrast, the microscopy images obtained with anti-phospho-tau 181 show that this antibody stains thick axonal extensions in the dentate *nucleus* and Bergmann glial cells surrounding the soma of Purkinje neurons in the cerebellar *cortex* more heavily. Thus, phospho-tau 217 seems to be a better biomarker than phospho-tau 181 for early neurotoxic amyloid production in the *cerebellum* of NPA-treated rats. Further experimental studies using higher resolution microscopy images, e.g., confocal microscopy images, of brain areas that are more sensitive to NPA degeneration (*striatum* and vicinal cortical premotor area) will be required to confirm the latter point.

In summary, our results reveal that kaempferol i.p. administration efficiently protects the rat *cerebellum* against NPA-induced microhemorrhages, overproduction of proinflammatory cytokines, activation of C3 complement protein, overproduction of neurotoxic Aβ(1-42) peptides and tau phosphorylation at 217 and 181 amino acid residues. The low toxicity of kaempferol for humans opens the possibility that kaempferol administration could be beneficial in the therapeutic treatment of NPA intoxication and related β-amyloidopathies and tauopathies. However, we wish to make a cautious note regarding the current limitations for the translational extrapolation of the results obtained in this work with kaempferol administration. First, the experimental design of this work involves preventive administration in an acute toxin model, which does not replicate the clinical conditions of chronic neurodegeneration. Second, studies dealing with brain exposure, pharmacokinetics, and safety margins for humans are necessary.

## 4. Materials and Methods

### 4.1. Chemicals

NPA and kaempferol were obtained from Sigma-Aldrich Spain (Sigma-Aldrich, St. Louis, MO, USA). Paraformaldehyde and glycerol were obtained from Panreac (Barcelona, Spain). Ketamine was obtained from Pfizer (Madrid, Spain). Atropine and diazepam were obtained from B. Braun (Rubí-Barcelona, Spain). The rest of the products were purchased from Sigma-Aldrich or Merck (Darmstadt, Germany), unless otherwise specified.

### 4.2. Animals and Treatments

In this work, we have followed protocols that were previously used in our laboratory for NPA and kaempferol systemic administration [10,12], which are summarized below.

During experiments, male Wistar rats, 9–10 weeks old, weighing 290–340 g, were housed in a 12 h light/dark cycle with free access to water and food. The experimental procedures were developed in accordance with the animal care guidelines of Council Directive 86/609/EEC of the European Union. The protocols were approved by the local government’s Animal Research Ethics Committee. The experimental animals were randomly allocated to the different experimental groups by the technical personnel of the animal facility of the university, i.e., observers not belonging to the research team for this work. All experimental animal handlings were performed under the supervision of the technical personnel of the animal facility of the university service, who were not involved in the research work, nor aware of the research goals of this work.

The rats were classified into three experimental groups: KNPA, NPA, and control. The selected number of rats was based on the inclusion of at least 6 specimens per group, guaranteeing a triplicate for each of the markers and/or antibodies used in the selected areas. The KNPA group (*n* = 6) was injected with a first dose of kaempferol solution, 21 mg/kg, 48 h before the start of the NPA treatment. From day 0–5 of treatment, a dose of 25 mg of NPA/kg body weight (b.w.) was injected i.p. every 12 h. Daily, 30 min before the morning NPA injection, another 21 mg/kg dose of kaempferol was administered to the rats. The NPA group (*n* = 6) was injected with 25 mg NPA/kg b.w. every 12 h over 5 days and, instead of kaempferol, received 1 mL injections with 2.4% *v*/*v* dimethyl sulfoxide (DMSO) in saline 48 h before NPA treatment and every day, 30 min before the morning NPA administration. The control group (*n* = 6) received 1 mL 2.4% *v*/*v* DMSO in saline (kaempferol vehicle) and 0.4 mL saline solution (NPA vehicle), with the same time schedule as the treatment groups. Systemic administration of NPA, at doses of 25 mg/kg of body weight every 12 h, caused marked behavioral alterations in rats, as we previously reported [10,12].

Motor impairment was assessed in all experimental animals throughout the experiment, similarly to what we previously did in our laboratory [10]. The animals were observed twice a day, just before the i.p. administration of NPA, and rated for the presence and severity of a variety of motor deficits, by means of a quantitative scale that has previously been described [64]. To avoid further animal loss on the fifth day of treatment, rats in this group with severe pathological symptoms (motor deficit ≥ 6 or weight loss ≥ 15%) were sacrificed at the end of day 4. KNPA group rats, as well as those in the control group, were treated until day 5 and then sacrificed.

Ketamine (50 μg/g), diazepam (2.5 μg/g) and atropine (0.05 μg/g) were applied at the end of the treatments to anesthetize the animals. The brains were immediately removed from the skull, washed in cold phosphate-buffered saline (PBS) pH 7.4, and then cut with a tissue slicer. In this work, cerebellar slices were used for 2,3,5-triphenyl tetrazolium chloride (TTC) staining, hematoxylin–eosin staining (H&E), Nissl staining, Congo Red staining, immunohistochemistry and Western blots.

### 4.3. Cerebellum Damage Monitored with TTC, H-E, Nissl and Congo Red Staining

Staining with TTC was performed as we previously described [10,25,65].

Coronal 1.5 mm-thick slices of *cerebellum* were taken from each of the three experimental groups (KNPA, NPA, and control), immersed in a 2% solution of TTC in PBS for 15 min at 37 °C, and observed under a Leica MZ APO stereomicroscope (Leica Microsystems, Heerbrugg, Switzerland).

For histological analysis, *cerebellum* samples were fixed in paraformaldehyde 4%, dehydrated, embedded in paraffin wax and sectioned in coronal sections at 7 μm. Vicinal sections were counter-stained with hematoxylin–eosin (H&E), Nissl (0.1% cresyl violet in 0.25% acetic acid) or Congo Red [43].

Sections were analyzed and digitally photographed in a Nikon Microscope Camera Digital Sight DS-Fi1 (Nikon Group Company, Tokyo, Japan) and Zeiss Axio imager 2 microscope (Carl Zeiss Microscopy GmbH, Jena, Germany), with a camera connected to a dedicated computer that was compatible with Image-Pro Plus software version ij154 (Media Cybernetics, Warrendale, PA, USA).

### 4.4. Cerebellum Samples Homogenization and Western Blotting of C3

*Cerebellum* samples were immediately frozen in liquid nitrogen and kept at −80 °C until use. Samples homogenization and Western blotting were performed as we previously described [11]. Briefly, weighed brain sections were homogenized at 0.14 g per mL in the following ice-cold buffer: 25 mM tris–(hydroxymethyl) aminomethane hydrochloride (Tris-HCl) at pH 7.4, 150 mM NaCl, 5 mM ethylenediaminetetraacetic acid, 50 mM NaF, 5 mM NaVO_3_ and 4-(1,1, 3,3-Tetramethylbutyl)phenyl-polyethylene glycol (Triton X-100) 0.25%, supplemented with the protease inhibitor cocktail SIGMAFAST S8820 (Sigma-Aldrich, Madrid, Spain). Aliquots of homogenized samples were transferred to an Eppendorf-type plastic vial and sonicated with 30–40 pulses of 100 w of 1 s, each using a titanium-tip sonicator in an ice-cold recipient. Then, the samples were centrifuged at 2000× *g* for 5 min at 4 °C, supernatants were collected, and their protein concentration was determined with Bradford’s method, using bovine serum albumin (BSA) as the protein standard. Supernatants supplemented with 40% glycerol were conserved at −80 °C until use for Western blotting. Sodium dodecyl sulfate-polyacrylamide gel electrophoresis (SDS-PAGE) has been performed in a BIO-RAD mini-Protean Tetra cell (Hercules, CA, USA), following a standard protocol with 7.5% acrylamide. Samples were loaded (20 μg of protein per lane) after the heat-denaturation of homogenate samples in 95 mM Tris-HCl buffer (pH 6.8), 3% sodium dodecyl sulfate (SDS), 1.5% *v*/*v* β-mercaptoethanol, 13% glycerol, and 0.005% bromophenol blue. SDS-PAGE gels were transferred to a polyvinylidene difluoride (PVDF) membrane of 0.2 μm average pore size in a standard transfer medium (Trans-BloT TransferMedium, BioRad, Hercules, CA, USA). PVDF membranes were blocked with 3% BSA, washed 6 times with Tris-buffered saline (TBS) supplemented with 0.05% Triton X-100 (TBST), and incubated with the primary antibody against the protein target for 1 h at room temperature with shaking. Thereafter, membranes were washed 6 times with TBST and incubated with the appropriate secondary antibody conjugated with horseradish peroxidase for 1 h at room temperature with shaking, washed 6 times with TBST, and treated with Clarity TM Western ECL Substrate, BIO-RAD. Western blot images were acquired and quantified with Bio-Rad ChemiDocTM XRS+. The primary antibody used in this work was the anti-C3 antibody (ab200999 –rabbit monoclonal, Abcam, Cambridge, UK, dilution 1:2000). After PVDF membrane image acquisition, the membranes were washed with deionized water, stripped, blocked with 3% BSA, and treated to quantify β-actin to monitor the protein load, as indicated above, using the mouse monoclonal anti-β-actin antibody (Sigma-Aldrich A1978, dilution 1:5000) or the polyclonal anti-β-actin antibody produced in rabbit (Sigma-Aldrich A5060, dilution 1:500) as the primary antibody, and anti-mouse or anti-rabbit IgG-Horseradish peroxidase (Sigma-Aldrich A0944 and A0545, respectively, dilution of 1:5000–1:10,000). After stripping, the signal of the staining with the primary antibodies against target protein C3 contributed less than 5% to the intensity of the β-actin band.

All the results were confirmed with Western blots of *n* = 6 different samples *per* experimental condition. Statistical analysis: the results of the Western blots are expressed as means ± standard error of the mean (SEM). Statistical analysis was carried out by Student’s *t*-test (independent two samples *t*-test). A significant difference was accepted at the *p* < 0.05 level.

### 4.5. Glial Fibrillary Acidic Protein (GFAP), TNFα, IL-1α, IL-1β, C1q and Component C3, β-Amyloid, Tau, p-Tau-217, and pTau-181 Immunohistochemistry

Vicinal coronal sections of cerebellum embedded in paraffin wax and cut 7 μm thick were selected to identify and localize different cells populations by using immunohistochemistry. We performed the next immunohistochemistry procedures.

#### 4.5.1. Glial Fibrillary Acidic Protein (GFAP), IL-1α, C1q, β-Amyloid and Tau

After blocking with 1% BSA for 30 min and incubation with 5% normal goat serum in 1% BSA and 0.1% Triton X-100 for 2 h, tissue sections were incubated with primary antibodies: dilution 1:400 for mouse anti-GFAP antibody (G3893, Sigma-Aldrich, Madrid, Spain); dilution 1:100 for both mouse anti-β-amyloid(1-42) antibody (A8354, Sigma-Aldrich) and mouse anti-Tau antibody (T46, ThermoFisher Scientific, Madrid, Spain, catalog nº 13-6400); and dilution 1:50 for both mouse anti-IL-1α antibody (sc-9983, Santa Cruz Biotechnology, Santa Cruz, CA, USA) and mouse anti-C1q-C antibody (Santa Cruz Biotechnology: sc-365301). All these antibodies are validated antibodies. Anti-Tau is validated in [66], and we have used the rest of the above-mentioned antibodies in previous publications [11,12,32].

Then, a secondary antibody (dilution 1:200) was added, a goat anti-mouse immunoglobulin G conjugated with alkaline phosphatase (IgG-AP), Santa Cruz Biotechnology: sc-3698. Finally, it was revealed with nitro blue tetrazolium/5-bromo-4-chloro-3-indolyl phosphate (NBT/BCIP), supplied by Roche, Darmstadt, Germany (catalog nº 1681451).

#### 4.5.2. Complement Component 3 (C3), TNFα, IL-1β, pTau-217 and pTau-181

Tissue sections were blocked with an endogenous avidin/biotin blocking kit (Abcam ab 64212) and incubated with primary antibodies: dilution 1:2000 for rabbit anti-C3 antibody (Abcam ab225539, a PBS-buffered version of ab200999, containing no BSA or sodium azide), dilution 1:250 for rabbit anti-IL-1β antibody (Abcam ab9722), and dilution 1:100 for rabbit anti-TNFα antibody (Abcam ab6671), rabbit anti-pTau-181 antibody (catalog nº 701530, Invitrogen/Thermo Fisher Scientific, Madrid, Spain) and rabbit anti-pTau-217 antibody (catalog nº 44-744, Invitrogen/Thermo Fisher Scientific). All these antibodies are validated antibodies. Anti-pTau-181 and anti-pTau-217 are validated in [67], and we have used the rest of the above-mentioned antibodies in previous publications [11,12]. Sections were incubated with avidin-biotinylated horseradish peroxidase complex (Vectastain ABC Kit PK6101). Chromogen development was performed with peroxidase substrate solution (Vector VIP substrate, SK-4600).

#### 4.5.3. Double Immunohistochemistry (GFAP + C3; GFAP + pTau-217; GFAP + pTau-181; β-Amyloid + C3; β-Amyloid + pTau217; β-Amyloid + pTau-181)

For double immunohistochemistry, primary antibody mouse anti-GFAP was applied together with rabbit anti-C3 or rabbit anti-pTau-217, or rabbit anti-pTau-181. Also, primary antibody mouse anti-β-amyloid was applied together with rabbit anti-C3, rabbit anti-pTau-217, or rabbit anti-pTau-181. Secondary antibodies, goat anti-mouse conjugated with alkaline phosphatase and biotinylated goat anti-rabbit Vectastain ABC Kit, were applied together. The chromogen development was carried out sequentially as follows: first, anti-GFAP or anti-β-amyloid and secondary antibody conjugated with alkaline phosphatase (blue) and, thereafter, the red color was developed with anti-C3 or anti-pTau-217 or anti-pTau-181 and a biotinylated secondary antibody conjugated with peroxidase.

Sections were analyzed and digitally photographed in a Nikon digital light DS-F1 and Zeiss Axio imager 2 microscope, with the camera controlled with a computer software that was compatible with Image-Pro Plus software (Media Cybernetics, Warrendale, PA, USA). Selected images of double immunohistochemistry β-amyloid + C3 (Figure 6) and GFAP + C3 (Supplementary Figure S3) were further processed with the “Split channels” tool in ImageJ^®^ version ij154 software, following the procedure outlined next: (1) transform the RGB image into a “Composite image”; (2) click “Split channels”; and (3) apply the “Invert” tool to the blue and red channel images, to convert the pixels stained with higher density in brighter pixels (for a direct positive correlation between pixel brightness and intensity of staining). All the paired blue and red channel images shown in this paper have been obtained with the same settings of minimum/maximum “Color balance” and 50% brightness.

### 4.6. Image Analysis

Quantitative densitometric analysis of microscopy images was performed using the ImageJ^®^ software. All microscopy images used for quantitative intensity analysis were acquired with the same microscope exposure time and brightness/contrast. In order to guarantee the reliability of densitometric analysis, the microscope exposure time was set as low as possible to minimize the presence of saturated staining pixels in the images selected for intensity quantitation. To this end, different batches of images were acquired for each experimental condition. Then, using the invert tool of the ImageJ^®^ software, we selected only the images displaying less than 2% saturated pixels in the histograms for quantitative intensity analysis. In addition, the inverted images were used for the analysis of the intensity of staining, due to two additional reasons: (1) inverted images directly correlate positive staining with higher values of intensity, and (2) the measure tool of the ImageJ^®^ software gives the minimum intensity *per* pixel in the regions of interest (ROI). The ROI selected for quantitative analysis of cerebellar *nuclei* positive staining have been those corresponding to the interposed (IP) and dentate *nuclei* (DN), accounting for a total number of pixels *per* frame ranging between 25,000 and 30,000. The lowest intensity pixels (the minimum values yielded by the measure tool in ImageJ^®^ software) in each field selected for analysis were used as a background or threshold. Thereafter, the background intensity was subtracted from the mean intensity of each ROI analyzed in this work.

### 4.7. Statistical Analysis

All data were obtained in at least three independent experiments, with replicates of three or more for each condition. Statistical analysis was carried out using Student’s *t*-test (independent two sample *t*-test) and results were expressed as the mean ± standard error of the mean (SEM). A significant difference was accepted at the *p* < 0.05 level and the *p*-values and 95% confidence interval (CI) for each experimental dataset are given in the legends for the corresponding figures.

## Data Availability

The original contributions presented in this study are included in the article/Supplementary Materials. Further inquiries can be directed to the corresponding authors.

## Supplementary Materials

The following supporting information can be downloaded at: https://www.mdpi.com/article/10.3390/ijms27062880/s1.

## Abbreviations

Aβ	Amyloid β peptides
APP	Amyloid β precursor protein
BSA	Bovine serum albumin
C1q	Complement protein component 1q
C3	Complement protein component 3
CI	95% confidence interval
DMSO	Dimethyl sulfoxide
GFAP	Glial fibrillary acidic protein
HD	Huntington’s Disease
H&E	Hematoxylin–eosin staining
IL-1α	Interleukin 1 alpha
IL-1β	Interleukin 1 beta
i.p.	Intraperitoneal
KNPA-rats	rats treated with i.p. co-administration of kaempferol and NPA
NF-κB	Nuclear Factor kappa B
NPA	3-Nitropropionic Acid
NPA-rats	rats treated with i.p. administration of NPA
PBS	Phosphate-buffered saline
pTau217	Phospho-tau (Thr217)
pTau181	Phospho-tau (Thr181)
PVDF	Polyvinylidene difluoride
SDS-PAGE	Sodium dodecyl sulfate-polyacrylamide gel electrophoresis
TBST	Tris-buffered saline (TBS) supplemented with 0.05% Triton X-100
TNFα	Tumor Necrosis Factor-alpha
TTC	2,3,5-Triphenyl tetrazolium chloride
