# Supplementary figures and images for: Kaempferol Protects Against Amyloid β Overproduction and the Rise of Phospho-Tau 217 and Phospho-Tau 181 in the Rat Cerebellum Induced by Acute 3-Nitropropionic Acid Administration
Source: Int J Mol Sci. 2026 Mar 22;27(6):2880. doi: 10.3390/ijms27062880 (PMC13026571; doi:10.3390/ijms27062880)

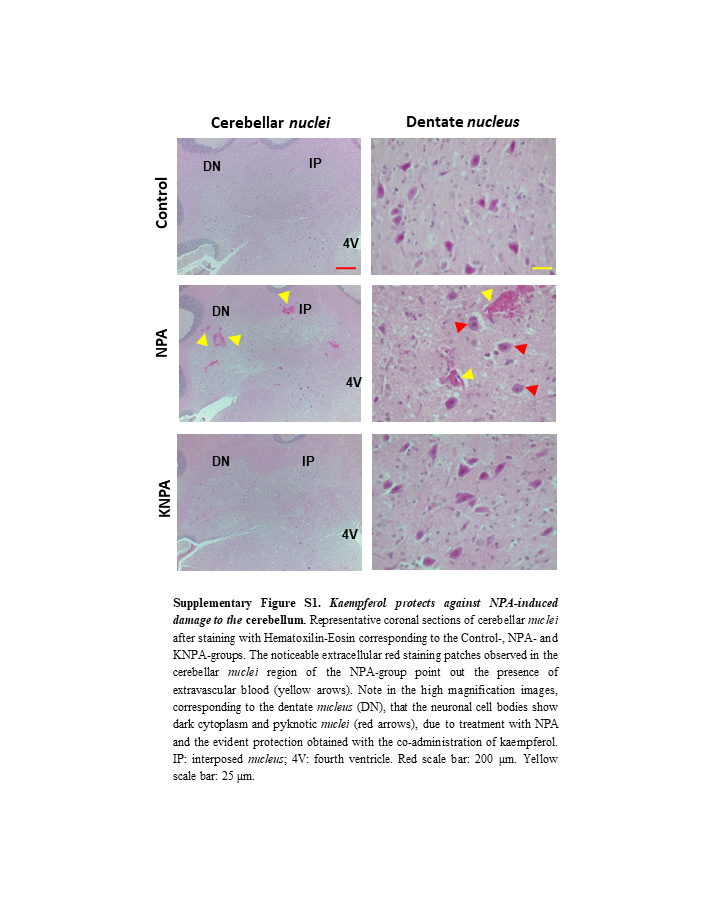

Supplement: Supplementary file 1 [file ijms-27-02880-s001.zip › ijms-4188278-supplementary/Figure S1.TIF]

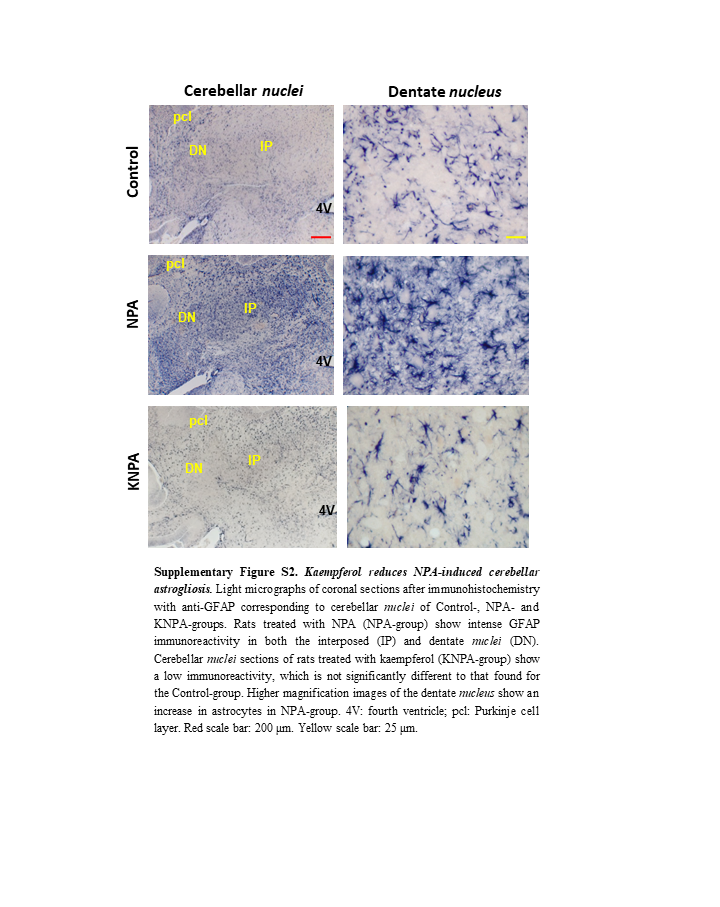

Supplement: Supplementary file 1 [file ijms-27-02880-s001.zip › ijms-4188278-supplementary/Figure S2.TIF]

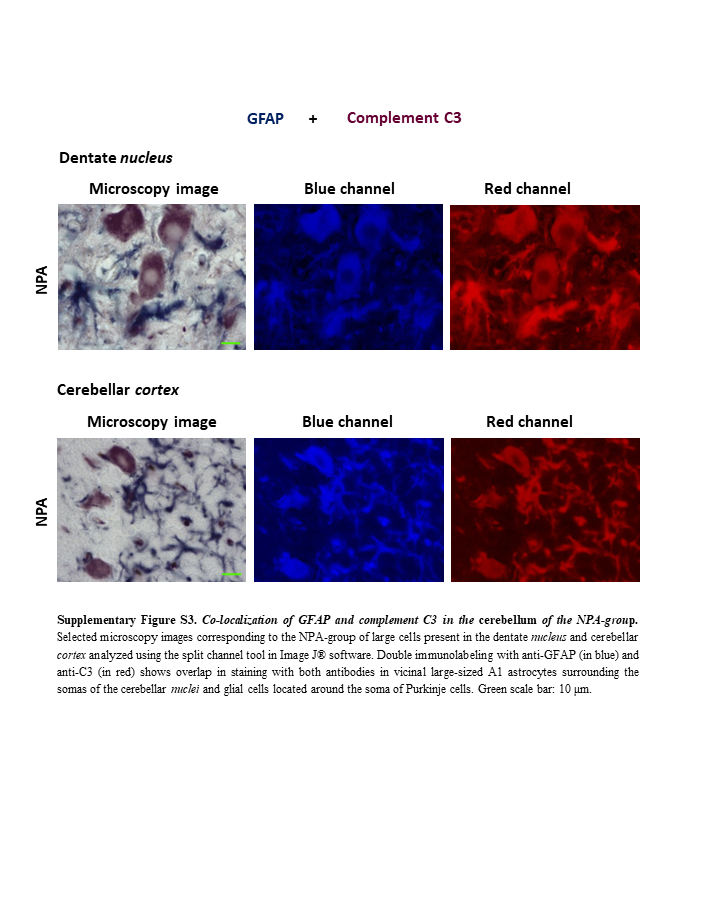

Supplement: Supplementary file 1 [file ijms-27-02880-s001.zip › ijms-4188278-supplementary/Figure S3.TIF]

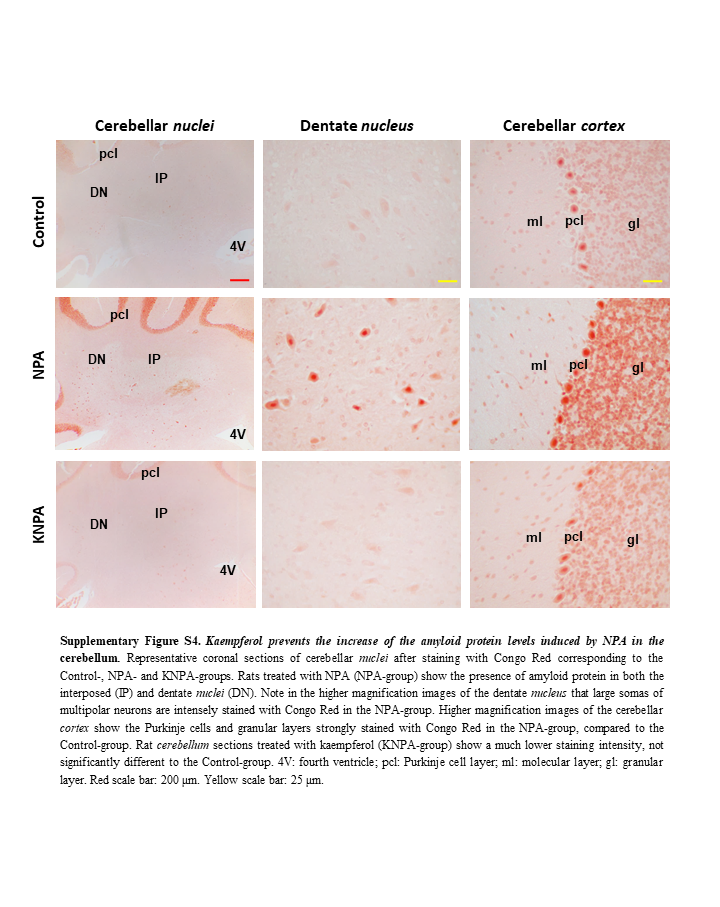

Supplement: Supplementary file 1 [file ijms-27-02880-s001.zip › ijms-4188278-supplementary/Figure S4.TIF]

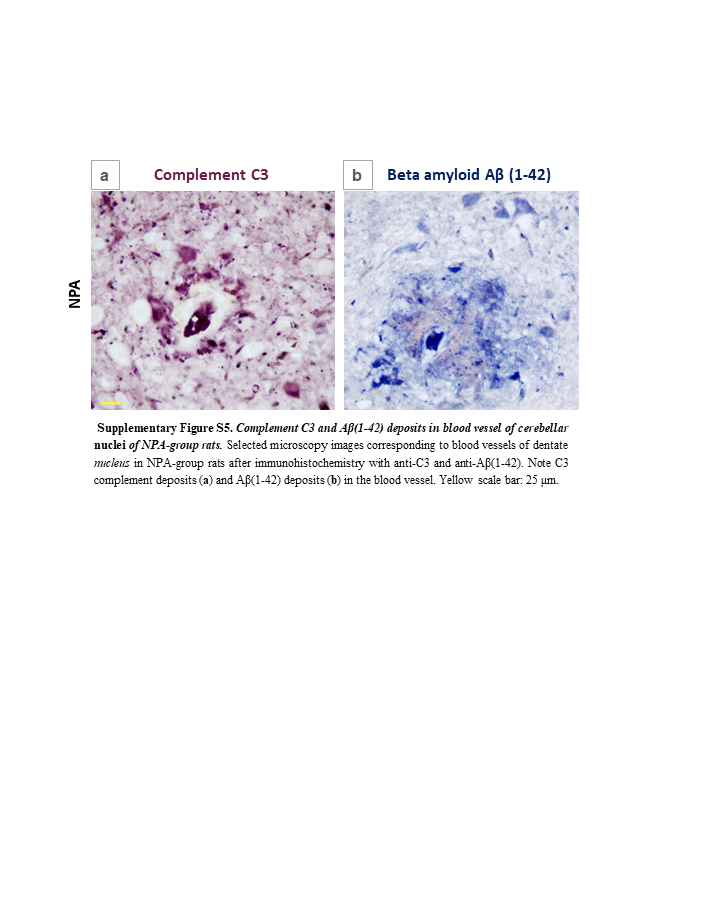

Supplement: Supplementary file 1 [file ijms-27-02880-s001.zip › ijms-4188278-supplementary/Figure S5.TIF]
